# Mitochondrial Protection by Astaxanthin Reduces Toxicity Caused by H_2_O_2_ and Doxorubicin in Human Cardiomyocytes

**DOI:** 10.3390/cells14221772

**Published:** 2025-11-12

**Authors:** Yulia Baburina, Aleksey Lomovsky, Yana Lomovskaya, Roman Sotnikov, Linda Sotnikova, Olga Krestinina

**Affiliations:** Institute of Theoretical and Experimental Biophysics, Russian Academy of Sciences, 142290 Pushchino, Russia

**Keywords:** human AC16 cardiomyocytes, astaxanthin, hydrogen peroxide, doxorubicin, mitochondrial dysfunction

## Abstract

Astaxanthin (AST) is a xanthophyll carotenoid known for its cardioprotective effects. In this study, we investigated the impact of AST on the survival of AC16 human cardiomyocytes under cardiotoxic conditions induced by hydrogen peroxide (H_2_O_2_) and doxorubicin (DOX). We assessed a series of parameters associated with cell death signaling, including: changes in cytosolic Ca^2+^ levels and reactive oxygen species (ROS) production; alterations in mitochondrial function (membrane potential ΔΨm and the content of key subunits of complexes I and II); and the levels of key apoptotic and ER stress markers. Our findings show that AST prevented the cytotoxic effects of both H_2_O_2_ and DOX. In the presence of AST, the number of viable cells increased, while Ca^2+^ levels, ROS production, and ΔΨm remained comparable to those in the control group. Furthermore, AST prevented the H_2_O_2_-induced decrease in the levels of the main subunits of respiratory chain complexes I and II. AST prevented the H_2_O_2_-induced increase in the levels of apoptotic caspases-8 and -3. It also protected against ER stress by counteracting the H_2_O_2_-mediated upregulation of BIP, CHOP, and ERO1α proteins. These results lead us to conclude that AST exerts a protective effect by inhibiting mitochondrial dysfunction.

## 1. Introduction

Cardiovascular diseases (CVDs) are among the most prevalent health issues and a leading cause of disability and mortality in many developed countries. Despite continuous scientific advancements and the introduction of new medical technologies for early diagnosis, a significant reduction in CVD-associated mortality has not been achieved, both in Russia and worldwide. Consequently, CVD remains a major social and medical challenge. Mitochondrial dysfunction is believed to play a pivotal role in the initiation and development of pathological changes characteristic of heart failure. This dysfunction manifests as decreased ATP production, impaired calcium homeostasis, and increased generation of reactive oxygen species (ROS), leading to oxidative stress [1]. Currently, considerable research attention is focused on preventing mitochondrial dysfunction using various antioxidants to enhance the body’s defense response and mitigate stress- and age-related oxidative damage [2]. Among natural antioxidants, carotenoids and their derivatives represent a large group of molecules naturally synthesized by plants and other photosynthetic organisms. These compounds can protect cells from light-mediated oxidative processes, free-radical peroxidation, and singlet oxygen damage [3]. Astaxanthin (AST, (3S,3′S)-3,3′-dihydroxy-β,β-carotene-4,4′-dione), a common xanthophyll carotenoid, stands out for its potent ability to quench oxygen radicals [2,4]. Numerous studies indicate that AST possesses multifaceted therapeutic and prophylactic activities, showing potential utility in various diseases, including diabetes, hyperlipidemia, steatohepatitis, neurodegenerative disorders, and cardiovascular diseases [2,5,6].

Numerous defects in the electron transport chain (ETC) complexes have been observed in various models of heart failure [7]. Impairments in respiratory complexes and ATP synthase can reduce ATP production and cause an accumulation of reducing equivalents such as NAD(P)H, which inhibit substrate oxidation and can lead to mitochondrial dysfunction [8]. Chronic administration of antioxidants, such as melatonin at pharmacological doses, has been found to improve mitochondrial function [9,10]. We recently demonstrated that oral melatonin administration had a protective effect on cardiac mitochondria isolated from rats treated with isoproterenol, an inducer of mitochondrial dysfunction [11]. Furthermore, we investigated the effect of AST on the viability of rat H9c2 cardiomyocytes in the presence of the cytotoxicity inducers H_2_O_2_ and DOX. A decrease in the mitochondrial membrane potential (ΔΨm) and an increase in ROS production during cell incubation with H_2_O_2_ and DOX led to the accumulation of dysfunctional mitochondria. The addition of AST to the cells increased ΔΨm and decreased ROS production, even in the presence of H_2_O_2_ and DOX [12].

Mitochondrial dysfunction impairs calcium buffering capacity, resulting in the disruption of cellular calcium homeostasis. This perturbation leads to aberrant calcium accumulation within the endoplasmic reticulum—a central organelle governing protein folding, trafficking, and intracellular calcium storage. Consequently, ER stress response pathways are activated, potentially initiating apoptotic signaling cascades and programmed cell death [13]. In addition, ER stress occurs when the ER is overloaded with misfolded proteins [14,15], a state that can contribute to various metabolic diseases. AST, as a potent antioxidant, has been reported to protect cells from ER stress and related disorders. Its protective effect is associated with an ability to alleviate ER stress and promote cell survival in various disease models [16,17,18]. The mechanisms underlying AST’s protective effects are multifaceted. For instance, its antioxidant properties help reduce oxidative stress, which can trigger ER stress. AST is also known to modulate the expression of genes and proteins involved in the unfolded protein response (UPR), such as BIP (binding immunoglobulin protein, or GRP78), a chaperone protein that aids in protein folding [19]. Additionally, ERO1α is a protein involved in oxidative protein folding within the ER [20]. The BIP-CHOP-ERO1α pathway represents a pro-apoptotic branch of the UPR that is activated by severe ER stress and typically leads to cell death [21]. Furthermore, AST can influence signaling pathways such as JNK and PI3K/Akt, which are involved in cell survival and apoptosis [22].

The experiments were conducted using the AC16 human cardiomyocyte line. This model consists of immortalized human cardiac muscle cells and is extensively employed **in vitro** studies for cardiovascular disease research. The aim of this work was to study the effect of AST on the survival of AC16 cardiomyocytes and on changes in cytosolic Ca^2+^ levels, ΔΨm, and ROS production. Additionally, we investigated changes in the content of the main subunits of complexes I and II of the electron transport chain (NDUFB8 and SDHB, respectively), proteins involved in apoptosis (Caspase-8 and -3), and proteins associated with ER stress and apoptosis (BIP, CHOP, and ERO1α) under these conditions.

## 2. Materials and Methods

**Cell Culture Methodology.** This study utilized human cardiomyocytes from the AC16 line, acquired from the American Type Culture Collection (ATCC, Manassas, VA, USA). The AC16 human cardiomyocyte cell line represents a proliferating cellular system established via fusion of primary cardiomyocytes isolated from adult human ventricular tissue with SV40-immortalized human fibroblasts possessing uridine auxotrophy. This cellular model retains fundamental cardiomyocyte characteristics, including expression of cardiac-specific molecular markers; sustained proliferative capacity in culture—a distinctive advantage over primary cardiomyocytes; and maintenance of cardiac functional properties across multiple passages. Cellular suspensions were plated in 96-well culture plates at a density of 5 × 10^3^ cells per well, with each well containing 100 µL of complete growth medium. The culture medium was composed of DMEM/F12 basal medium (Sigma-Aldrich, St. Louis, MO, USA) supplemented with 12.5% (*v*/*v*) fetal bovine serum (Gibco, Waltham, MA, USA) and 40 µg/mL gentamicin sulfate (Sigma-Aldrich, St. Louis, MO, USA). Cells were maintained for 24 h at 37 °C in a humidified incubator with 5% CO_2_. All experimental procedures were initiated 24 h post-seeding.

**Assessment of Cytotoxicity.** Cellular viability was evaluated following 24 h exposure to astaxanthin (AST; Macklin, Shanghai, China; 0–100 µM), hydrogen peroxide (H_2_O_2_; Sigma-Aldrich, St. Louis, MO, USA; 1–400 µM), or doxorubicin (DOX; Sigma-Aldrich, St. Louis, MO, USA; 1–400 µM). Viability was quantified as a percentage relative to untreated control cultures using a resazurin reduction assay. The metabolic activity of viable cells was determined by measuring the conversion of resazurin to resorufin (Sigma-Aldrich, St. Louis, MO, USA). Cells were incubated with 30 µg/mL resazurin for 4 h at 37 °C under 5% CO_2_ atmosphere. Fluorescence intensity was recorded using an Infinite F200 microplate reader (Tecan, Männedorf, Switzerland) with excitation at 532 nm and emission at 590 nm.

**Assessment of Intracellular Calcium Dynamics in AC16 Cardiomyocytes.** Intracellular calcium levels were quantified using the fluorescent calcium indicator Fluo-4 AM (Sigma-Aldrich, St. Louis, MO, USA). Cells were plated in 96-well plates at a density of 5 × 10^3^ cells/well and treated with experimental compounds for 24 h. Following treatment, cells were harvested by centrifugation (250× *g*, 5 min, 25 °C), washed with phosphate-buffered saline (PBS), and resuspended in PBS to a final concentration of 5 × 10^3^ cells/mL. Cell suspensions were incubated with 2 µM Fluo-4 AM for 30 min under standard culture conditions (37 °C, 5% CO_2_). After dye loading, cells were washed with PBS to remove excess fluorophore. Fluorescence measurements were performed using an Infinite F200 PRO microplate reader (Tecan, Männedorf, Switzerland) with excitation at 494 nm and emission detection at 516 nm.

**Assessment of Mitochondrial Membrane Potential (ΔΨm)**. Mitochondrial membrane potential was evaluated using the potentiometric fluorescent dye 3,3′-dihexyloxacarbocyanine iodide (DiOC_6_(3); Sigma-Aldrich, St. Louis, MO, USA). Cells were plated in 96-well plates at a density of 5 × 10^3^ cells/well. Cells were loaded with 10 nM DiOC_6_(3) and incubated for 30 min at 37 °C under 5% CO_2_ atmosphere. Following dye loading, cells were washed with phosphate-buffered saline (PBS) to remove unincorporated probe. Fluorescence intensity was quantified using an Infinite F200 PRO microplate reader (Tecan, Männedorf, Switzerland) with excitation at 485 nm and emission at 530 nm. Results were normalized and expressed as percentage values relative to untreated control cells.

**Assessment of Intracellular Reactive Oxygen Species Generation**. Intracellular ROS levels were quantified using the fluorogenic probe 2′,7′-dichlorodihydrofluorescein diacetate (DCFH-DA; Sigma-Aldrich, St. Louis, MO, USA). Cells were plated in 96-well plates at a density of 5 × 10^3^ cells/well. Cellular staining was performed with 50 µM DCFH-DA during a 30 min incubation period at 37 °C under 5% CO_2_ conditions. Subsequent to probe loading, cells underwent a single wash cycle with phosphate-buffered saline (PBS, Sigma-Aldrich, St. Louis, MO, USA) to remove excess dye. Fluorescence measurements were conducted using an Infinite F200 PRO microplate reader (Tecan, Männedorf, Switzerland) with optimal excitation at 485 nm and emission detection at 530 nm. Experimental results were normalized and expressed as percentage values relative to untreated control groups.

**Protein Electrophoresis and Immunoblot Analysis.** Cellular treatments included AST (5 and 10 µM), H_2_O_2_ (100 µM), and DOX (5 µM). Post-treatment, cells were washed with ice-cold PBS and pelleted by centrifugation (1500× *g*, 3 min, RT). Cell lysis was performed using RIPA buffer containing protease and phosphatase inhibitors, followed by 1 h rotation at 4 °C and centrifugation (13,000× *g*, 10 min). Protein quantification was conducted via Bradford assay [23]. Lysates were denatured in Laemmli buffer (Bio-Rad, Hercules, CA, USA) at 95 °C for 5 min and resolved on 12.5% SDS-polyacrylamide gels. Proteins were electrophoretically transferred to 0.2 µm nitrocellulose membranes (Bio-Rad, Hercules, CA, USA). Membranes were blocked with Roti-Block (Carl Roth GmbH + Co., Karlsruhe, Germany) for 1 h at RT and probed with primary antibodies against: OXPHOS complex subunits (dilution 1:1000, Abcam, Cambridge, UK, ab110413), CHOP (dilution 1:1000, FineTest, Wuhan, China, FNab01667), BIP (dilution 1:1000, Affinity Biosciences, Cincinnati, OH, USA, AF0729), Caspase-3 (dilution 1:1000, BD Biosciences, Franklin Lakes, CA, USA, 610322), Caspase-8 (dilution 1:500, ENZO, Carmel-by-the-Sea, CA, USA, ALX-804-242), and ERO1α (dilution 1:100, FineTest, Wuhan, China, FNab02852) following manufacturers’ protocols. GAPDH (dilution 1:1000, Santa Cruz, CA, USA, sc-47724) and β-tubulin (dilution 1:1000, Cell Signaling, Danvers, MA, USA, #2146) served as loading controls. Immunoreactive bands were visualized using ECL detection on a ChemiDoc Touch Imaging System (Bio-Rad, Hercules, CA, USA).

**Statistical analysis.** All data were subjected to one-way analysis of variance (ANOVA) with subsequent **post hoc** comparisons using the Student-Newman-Keuls method. Statistical significance was defined as *p* < 0.05 for all analyses.

## 3. Results

**Figure 1:**
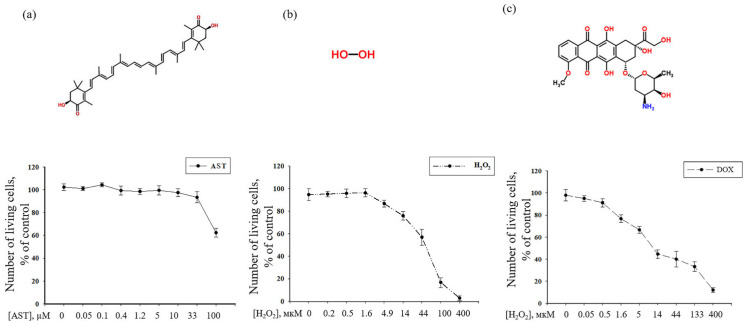
Dose–response effects of astaxanthin, hydrogen peroxide, and doxorubicin on AC16 cell viability. Viability quantification was performed 24 h after compound exposure using resazurin metabolism assay. (**a**) Upper panel: Chemical structure of astaxanthin (AST). Lower panel: AC16 cardiomyocytes following treatment with AST (0–100 μM). (**b**) Upper panel: Structural formula of hydrogen peroxide (H_2_O_2_). Lower panel: Cellular response to H_2_O_2_ exposure (0–400 μM). (**c**) Upper panel: Molecular structure of doxorubicin (DOX). Lower panel: Morphological assessment of cells treated with DOX (0–400 μM). Results are expressed as percentage of untreated control (defined as 100%) and shown as mean ± SD (n = 10 independent experiments).

In the present study, we demonstrated the protective effect of AST using models where cytotoxicity was induced in human AC16 cardiomyocytes by hydrogen peroxide (H_2_O_2_) [24] and doxorubicin (DOX) [25]. The AC16 cardiomyocyte line is a proliferating human cell line obtained by fusing primary cells from adult human cardiac ventricular tissue, which can be used to study cardiomyocyte developmental regulation [26]. To determine the cytotoxic concentrations of AST, H_2_O_2_, and DOX, cells were incubated with a range of compound concentrations (Figure 1). As shown in Figure 1a, AST concentrations up to 33 µM did not affect cell viability compared to the control. However, at 100 µM, cell viability decreased by 40%, indicating AST toxicity at higher concentrations. The minimum concentration of H_2_O_2_ that caused an approximately 20% decrease in cell viability was 14 µM; viability decreased by approximately 45% at 44 µM H_2_O_2_ (Figure 1b). At 400 µM H_2_O_2_, cell viability was nearly abolished. Investigation of the effect of different DOX concentrations showed that a minimum concentration of 1.6 µM decreased viability by approximately 25%, while 14 µM and 400 µM DOX reduced viability by approximately 55% and 90%, respectively (Figure 1c). Our initial results revealed that among the tested concentrations of AST (5–100 µM), only the 5 µM and 10 µM doses conferred significant protection against both H_2_O_2_- and doxorubicin-induced cytotoxicity. Consequently, these two concentrations were selected for the present study.

**Figure 2:**
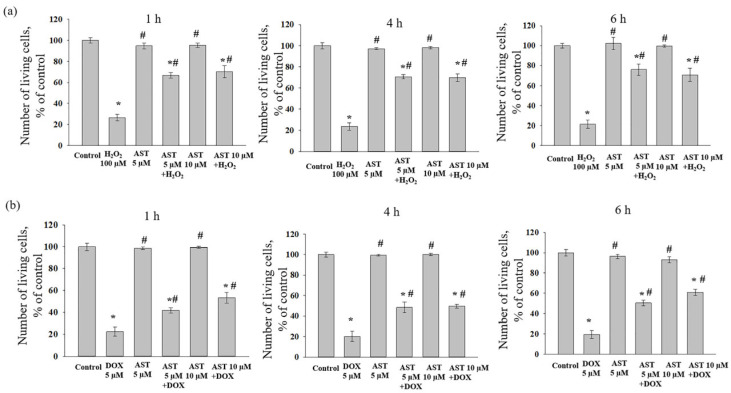
Viability of AC16 cardiomyocytes treated with AST, H_2_O_2_, and DOX. Dependence of AC16 cardiomyocyte viability on the concentration of AST (5 and 10 µM), H_2_O_2_ (100 µM), and DOX (5 µM). (**a**) Cytotoxic effect of AST and H_2_O_2_ during 1, 4, and 6 h incubation with AST. (**b**) Cytotoxic effect of AST and DOX during 1, 4, and 6 h incubation with AST. The number of viable cells in the untreated control culture was set as 100%. Data are presented as mean ± SD (n = 10). * *p* < 0.05 indicates a significant effect compared to the control (untreated culture); # *p* < 0.05 indicates a significant effect compared to H_2_O_2_ or DOX treatment alone.

The combined effect of AST, H_2_O_2_, and DOX on AC16 cell viability was assessed after one, four, and six hours of incubation, as the change in viability depended on both the substance concentration and the incubation time with AST (Figure 2). Incubation of cardiomyocytes with AST (5 and 10 µM) for 1, 4, and 6 h did not affect cell viability compared to the control. Treatment with H_2_O_2_ (100 µM) alone decreased viability by an average of 76% relative to the control (Figure 2a). When cells were pre-incubated with 5 µM AST for 1, 4, and 6 h followed by the addition of 100 µM H_2_O_2_, cell viability decreased by approximately 32%, 30%, and 24%, respectively, compared to the control. Pre-incubation with 10 µM AST under similar conditions resulted in viability decreases of 30%, 31%, and 30% relative to the control.

Treatment with 5 µM DOX alone decreased AC16 viability by 80% compared to the control (Figure 2b). When DOX was added to AST-pre-incubated cells, viability decreased by 58% (1 h incubation with 5 µM AST), 46.5% (1 h incubation with 10 µM AST), 52% (4 h incubation with 5 and 10 µM AST), 49.5% (6 h incubation with 5 µM AST), and 39% (6 h incubation with 10 µM AST) compared to the control. Although the addition of cytotoxicity inducers to AST-pre-incubated cells reduced viability relative to the control, the number of viable cells was significantly higher than with the inducers alone. Based on these results, an AST concentration of 5 and 10 µM and a pre-incubation time of 4 h were selected for subsequent studies measuring cytosolic Ca^2+^, mitochondrial membrane potential, and ROS production, prior to the addition of 100 µM H_2_O_2_ or 5 µM DOX. Although the impact of higher AST concentrations and prolonged pre-incubation periods on cardiomyocyte viability was assessed, no cytoprotective effect of AST was detected. Based on these preliminary results, AST concentrations of 5 and 10 µM were selected for use with 100 µM H_2_O_2_ or 5 µM DOX in this study.

Calcium ions (Ca^2+^) act as crucial secondary messengers involved in numerous cellular processes, including protein synthesis, gene expression, cell cycle progression, metabolism, and apoptosis [27].

**Figure 3:**
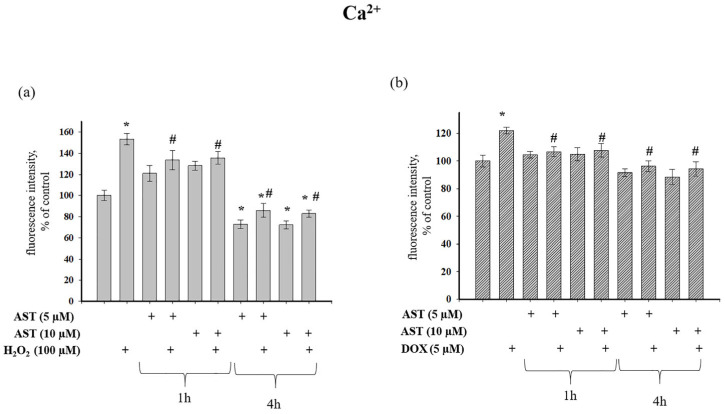
Modulation of cytosolic Ca^2+^ levels by astaxanthin in H_2_O_2_- and doxorubicin-treated AC16 cardiomyocytes. Cells were pre-treated with AST (5 or 10 µM) for 1 or 4 h prior to exposure to cytotoxic concentrations of H_2_O_2_ (100 µM) or DOX (5 µM). (**a**) Intracellular Ca^2+^ concentrations following AST and H_2_O_2_ co-treatment. (**b**) Intracellular Ca^2+^ levels after AST and DOX administration. Fluorescence intensity values were normalized to untreated controls. Data represent mean ± SD from six independent experiments (n = 6). Asterisks denote significant differences compared to control (* *p* < 0.05), while hash symbols indicate significant effects relative to H_2_O_2_ or DOX treatment alone (# *p* < 0.05).

We next investigated the effect of AST on cytosolic Ca^2+^ levels in AC16 cardiomyocytes under stress induced by H_2_O_2_ and DOX (Figure 3). Figure 3a shows that H_2_O_2_ increased cytosolic Ca^2+^ by 53% relative to control values. Pre-incubation of cells with 5 and 10 µM AST for 1 h increased cytosolic Ca^2+^ by 21% and 28%, respectively. The combined application of AST and H_2_O_2_ after 1 h pre-incubation increased Ca^2+^ levels by 33% and 35%, respectively. In contrast, a 4 h pre-incubation with 5 and 10 µM AST decreased cytosolic Ca^2+^ by 28% and 30%, respectively. Subsequent addition of H_2_O_2_ to these cells decreased cytosolic Ca^2+^ by 14% and 20% compared to the control, representing a 1.77 and 1.86-fold reduction compared to the effect of H_2_O_2_ alone. Figure 3b shows that 5 µM DOX increased cytosolic Ca^2+^ by 22% relative to the control. A 1 h pre-incubation with AST, both alone and in combination with DOX, maintained cytosolic Ca^2+^ at control levels. However, a 4 h pre-incubation with 5 and 10 µM AST decreased cytosolic Ca^2+^ by 10% and 12% compared to the control, and by 25% and 27% relative to DOX alone.

**Figure 4:**
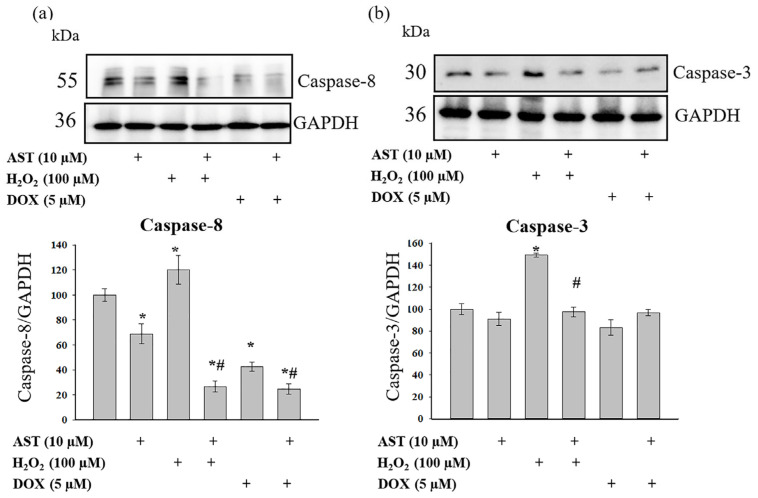
AST modulation of caspase-8 and caspase-3 expression in H_2_O_2_- and doxorubicin-stressed AC16 cardiomyocytes. Cells were pre-treated with 10 µM AST for 4 h prior to induction of cytotoxicity with either 100 µM H_2_O_2_ or 5 µM DOX. (**a**) Upper panel: representative immunoblot depicting caspase-8 protein expression. Lower panel: densitometric quantification of caspase-8 levels normalized to GAPDH. (**b**) Upper panel: representative immunoblot showing caspase-3 expression. Lower panel: quantitative analysis of caspase-3 protein levels relative to GAPDH. Untreated control values were set as 100%. Data represent mean ± standard deviation from three independent experiments (n = 3). Asterisks indicate statistically significant differences compared to control (* *p* < 0.05), while hash symbols denote significant effects relative to H_2_O_2_ or DOX treatment alone (# *p* < 0.05).

Cytosolic Ca^2+^, caspase-8, and caspase-3 are key components of the apoptotic pathway. Caspase-8 acts as an initiator, caspase-3 is a key effector caspase, and Ca^2+^ serves as a regulator and trigger for these events [28]. Therefore, we investigated the effect of AST on changes in caspase-8 and caspase-3 levels under induced stress (Figure 4).

Figure 4 presents Western blots probed with antibodies against caspase-8 (Figure 4a) and caspase-3 (Figure 4b), along with quantitative densitometry analysis. AST alone reduced caspase-8 levels by 25%, while caspase-3 levels remained unchanged. H_2_O_2_ treatment increased both caspase-8 and caspase-3 levels by 20% and 50%, respectively. The combined treatment of AST and H_2_O_2_ decreased caspase-8 levels by 65%, while caspase-3 levels were comparable to the control. Relative to the control, DOX reduced caspase-8 and caspase-3 levels by 60% and 15%, respectively. AST co-treatment further suppressed caspase-8 expression (75% reduction) but did not alter caspase-3 levels beyond control values.

**Figure 5:**
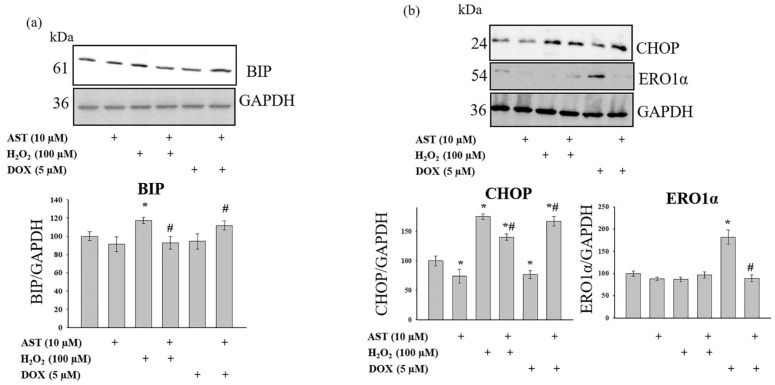
Effect of AST on BIP, CHOP, and ERO1α levels under H_2_O_2_- and DOX-induced cytotoxicity in AC16 cardiomyocytes. Cells were pre-incubated with 10 µM AST for 4 h. Cytotoxicity was induced with 100 µM H_2_O_2_ or 5 µM DOX. (**a**) Upper panel: representative immunoblot for BIP. Lower panel: quantitative analysis of protein levels normalized to GAPDH. (**b**) Upper panel: representative immunoblots for CHOP and ERO1α. Lower panel: quantitative analysis of protein levels normalized to GAPDH. The protein level in the untreated cell lysate was set as 100%. Data are presented as mean ± SD (n = 3). * *p* < 0.05 indicates a significant effect compared to the control; # *p* < 0.05 indicates a significant effect compared to H_2_O_2_ or DOX treatment alone.

Key components of the ER stress response include the binding immunoglobulin protein (BIP, also known as GRP78) and the C/EBP homologous protein (CHOP). BIP is involved in the initial adaptive phase, while CHOP can promote protective responses or apoptosis under severe or prolonged stress [29]. We examined changes in BIP, CHOP, and ERO1α levels under our experimental conditions (Figure 5).

Figure 5a shows Western blots for BIP, and Figure 5b shows blots for CHOP and ERO1α. AST alone did not alter BIP levels but reduced CHOP and ERO1α levels by 26% and 11%, respectively. H_2_O_2_ treatment increased BIP and CHOP levels by 17% and 75%, respectively, and decreased ERO1α by 12%. The combination of AST and H_2_O_2_ restored BIP to control levels. Under these conditions, CHOP levels were 40% higher than the control but 20% lower than with H_2_O_2_ alone. ERO1α levels were unchanged compared to H_2_O_2_ alone. DOX treatment did not alter BIP levels but decreased CHOP by 23% and increased ERO1α by 81%. The combination of AST and DOX did not change BIP or ERO1α levels compared to the control but decreased CHOP by 78%.

**Figure 6:**
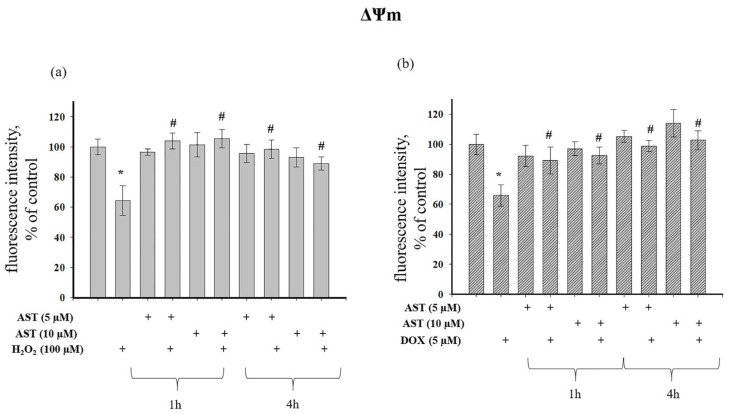
Effect of AST on mitochondrial membrane potential (ΔΨm) under H_2_O_2_- and DOX-induced cytotoxicity in AC16 cardiomyocytes. Cells were pre-incubated with 5 and 10 µM AST for 1 or 4 h. Cytotoxicity was induced with 100 µM H_2_O_2_ or 5 µM DOX. (**a**) Changes in ΔΨm in the presence of AST and H_2_O_2_. (**b**) Changes in ΔΨm in the presence of AST and DOX. The fluorescence intensity of the untreated cell culture was used as a control. Data are presented as mean ± SD (n = 6). * *p* < 0.05 indicates a significant effect compared to the control; # *p* < 0.05 indicates a significant effect compared to H_2_O_2_ or DOX treatment alone.

Cytosolic Ca^2+^ and mitochondrial membrane potential (ΔΨm) are closely linked, with changes in one affecting the other. The interaction between cytosolic Ca^2+^ accumulation and ΔΨm is a dynamic process critical for regulating cellular energy production [30]. We investigated the effect of AST on ΔΨm changes during H_2_O_2_- and DOX-induced cytotoxicity (Figure 6). Figure 6a shows that H_2_O_2_ decreased ΔΨm by 36% relative to the control. ΔΨm was unaffected by 1 h or 4 h incubation with AST alone. When H_2_O_2_ was added to AST-pre-incubated cells, AST exhibited a protective effect, as ΔΨm values were close to control levels and significantly higher than with H_2_O_2_ alone. DOX treatment decreased ΔΨm by 35% (Figure 6b), while AST pre-incubation alone had no effect. The combination of AST and DOX maintained ΔΨm at control levels, regardless of pre-incubation time, and significantly higher than with DOX alone, demonstrating a protective effect of AST.

Figure 6a shows that H_2_O_2_ caused a 36% decrease in ΔΨm in cells relative to the control. ΔΨm did not change with 1 and 4 h incubation of cells with AST. When H_2_O_2_ was added to AST-incubated cells, a protective effect of AST was observed, since the ΔΨm value was close to the control. However, compared to the single effect of H_2_O_2_, an increase in ΔΨm was observed at both AST concentrations and regardless of the time of cell incubation with AST. The addition of DOX to cells resulted in a 35% decrease in ΔΨm compared to the control, whereas 1 and 4 h incubation of cells with AST (5 and 10 μM) did not change ΔΨm in cells (Figure 6b). With the combined use of AST and DOX, the ΔΨm value did not differ from the control, regardless of the time of pre-incubation with AST, but increased relative to the single effect of DOX. AST exhibited a protective effect, maintaining ΔΨm at the level of control values despite the presence of DOX in the cells.

**Figure 7:**
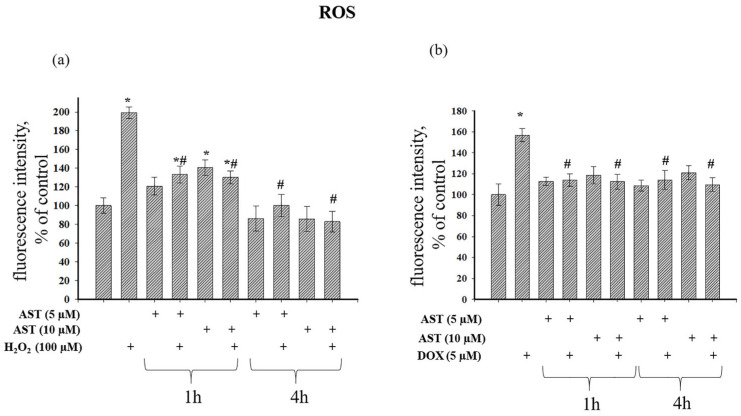
Effect of AST on intracellular ROS production under H_2_O_2_- and DOX-induced cytotoxicity in AC16 cardiomyocytes. Cells were pre-incubated with 5 and 10 µM AST for 1 or 4 h. Cytotoxicity was induced with 100 µM H_2_O_2_ or 5 µM DOX. (**a**) Changes in intracellular ROS production in the presence of AST and H_2_O_2_. (**b**) Changes in intracellular ROS production in the presence of AST and DOX. The fluorescence intensity of the untreated culture was used as a control. Data are presented as mean ± SD (n = 6). * *p* < 0.05 indicates a significant effect compared to the control; # *p* < 0.05 indicates a significant effect compared to H_2_O_2_ or DOX treatment alone.

An elevated ΔΨm can lead to electron leakage in the ETC, increasing ROS generation. Conversely, oxidative stress caused by ROS can depolarize ΔΨm [31]. Figure 7 shows the effect of AST on ROS production during H_2_O_2_- and DOX-induced cytotoxicity. H_2_O_2_ alone increased ROS nearly 2-fold (Figure 7a).

A 1 h incubation with 5 and 10 µM AST increased ROS by 20% and 40%, respectively. The combined application of AST and H_2_O_2_ after 1 h increased ROS by 33% and 30% compared to the control, but this represented a 33% and 35% reduction compared to H_2_O_2_ alone. A 4 h incubation with 5 and 10 µM AST decreased ROS production by 14% and 15%, respectively. The combined treatment of 5 µM AST and H_2_O_2_ after 4 h did not alter ROS relative to the control but reduced it by 50% compared to H_2_O_2_ alone. The combination of 10 µM AST and H_2_O_2_ decreased ROS by 17% compared to the control and by 58.3% relative to H_2_O_2_ alone. Figure 7b shows that 5 µM DOX increased ROS production by 57%. A 1 h pre-incubation with AST did not affect basal ROS, and its combination with DOX maintained ROS at control levels, representing a 21.8% and 30% reduction compared to DOX alone. A 4 h pre-incubation with AST also did not affect basal ROS, and its combination with DOX kept ROS at control levels, representing a 27% and 31% reduction compared to DOX alone.

**Figure 8:**
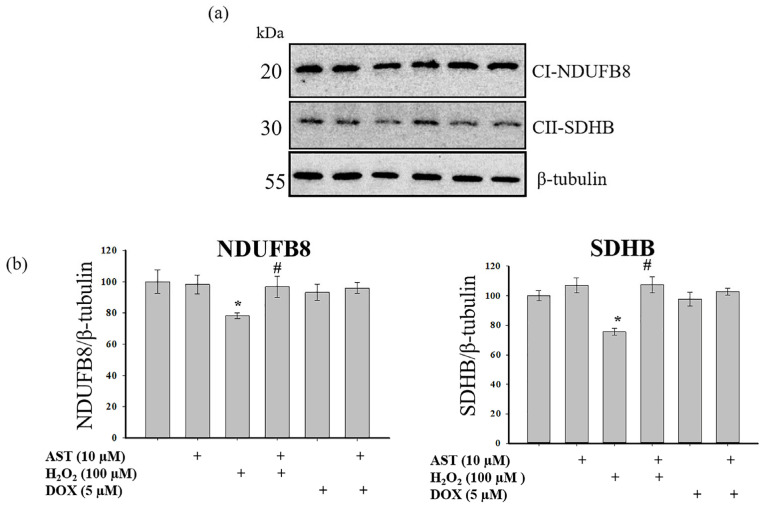
Effect of AST on the levels of NDUFB8 and SDHB subunits under H_2_O_2_- and DOX-induced cytotoxicity in AC16 cardiomyocytes. Cells were pre-incubated with 10 µM AST for 4 h. Cytotoxicity was induced with 100 µM H_2_O_2_ or 5 µM DOX. (**a**) Representative immunoblots for the NDUFB8 (Complex I) and SDHB (Complex II) subunits. β-tubulin was used as a loading control. (**b**) Quantitative assessment of immunoblots performed using computer densitometry. The protein level in the untreated cell lysate was set as 100%. Data are presented as mean ± SD (n = 3). * *p* < 0.05 indicates a significant effect compared to the control; # *p* < 0.05 indicates a significant effect compared to H_2_O_2_ treatment alone.

A primary mechanism underlying mitochondrial dysfunction in various pathologies is the disruption of multiprotein complexes in the mitochondrial respiratory chain, which serve as electron carriers [32]. We analyzed changes in the content of the main subunits of respiratory chain complexes I and II (Figure 8). Figure 8a shows Western blots for the NDUFB8 subunit of complex I and the SDHB subunit of complex II. Figure 8b shows quantitative densitometry analysis normalized to β-tubulin.

A 4 h incubation with AST alone did not alter subunit levels. H_2_O_2_ treatment decreased the levels of both subunits by approximately 25%. However, pre-incubation with 10 µM AST prevented this decrease, maintaining subunit levels at control values and significantly higher than with H_2_O_2_ alone. Treatment with 5 µM DOX alone, or in combination with AST, did not significantly alter the levels of NDUFB8 or SDHB compared to the control.

## 4. Discussion

Mitochondria are recognized as the primary organelles responsible for cellular ATP production. These organelles are particularly vulnerable to oxidative damage, and the associated structural alterations become more pronounced under pathological conditions [33]. Mitochondrial dysfunction is a common feature in various diseases. For instance, impaired oxygen supply can disrupt mitochondrial function and ATP synthesis, leading to a significant decline in cardiac ATP production during myocardial ischemia [34]. To mitigate the oxidative damage inherent in the pathogenesis of various heart diseases, considerable research efforts are directed at harnessing antioxidants to bolster the body’s endogenous defense systems. Astaxanthin (AST), a potent antioxidant, has been shown to inhibit oxidative stress-induced mitochondrial dysfunction in living cells [34,35].

Previous studies have demonstrated that AST prevents isoproterenol-induced mitochondrial dysfunction in rat heart mitochondria [36,37], and ameliorates heat stress-induced impairment of blastocyst development by increasing ΔΨm [38]. In H9c2 rat cardiomyocytes, AST reduced cytosolic Ca^2+^ levels and ROS production, while increasing ΔΨm, thereby enhancing cell survival [12,39]. Consistent with these findings, our present study shows that under H_2_O_2_- and DOX-induced cytotoxicity in human AC16 cardiomyocytes, AST prevented the increase in cytosolic Ca^2+^ and ROS production, attenuated the decrease in ΔΨm, and consequently increased the number of viable cells.

Caspase-3 and caspase-8 activities are known to be modulated by cytosolic Ca^2+^ levels. Caspase-3 activation can be triggered by an increase in cytosolic Ca^2+^, but it can also be a cause of elevated Ca^2+^ [40]. Calcium can directly activate caspase-3 [41], while its effect on caspase-8 is often indirect, involving the modulation of protein–protein interactions and upstream signaling pathways [42]. Furthermore, elevated caspase-3 levels are a hallmark of apoptosis [43]. In our study, we observed increased cytosolic Ca^2+^ levels following H_2_O_2_- and DOX-induced cytotoxicity. This could potentially lead to increased caspase-8 levels and subsequent caspase-3 activation. However, we did not detect activated caspase-3, as no cleavage was observed. This suggests that the apoptotic pathways were likely in their initial stages. Interestingly, despite the increase in cytosolic Ca^2+^ induced by both H_2_O_2_ and DOX, significant changes in caspase-8 and -3 levels were observed only with H_2_O_2_ treatment, indicating distinct mechanisms of action for these two inducers. AST reduced the levels of caspase-8 and -3 in the presence of H_2_O_2_, correlating with the normalization of cytosolic Ca^2+^ and improved cell survival.

The primary ER chaperone protein, BIP, interacts with Ca^2+^ in the ER lumen and cytosol, thereby influencing Ca^2+^ homeostasis and protein folding. BIP binds Ca^2+^ and contributes to its storage within the ER [44]. During ER stress, the transcription factor CHOP (C/EBP homologous protein) is upregulated [45]. Our results demonstrate that both H_2_O_2_ and DOX induced an increase in cytosolic Ca^2+^ and ROS production, accompanied by a collapse in mitochondrial membrane potential, unequivocally indicating mitochondrial dysfunction. Under H_2_O_2_-induced ER stress, we detected an increase in BIP and CHOP levels, but not in ERO1α. In contrast, DOX treatment did not alter BIP or CHOP levels but significantly increased ERO1α. Pre-incubation with AST reduced the levels of BIP and CHOP during H_2_O_2_-induced cytotoxicity, while ERO1α levels remained unchanged. Conversely, the addition of DOX to AST-pre-incubated cells increased BIP and CHOP levels compared to DOX alone but decreased ERO1α under these conditions. The differential changes in these protein levels likely reflect the engagement of distinct signaling pathways by H_2_O_2_ and DOX.

Complex I (NADH dehydrogenase) of the mitochondrial respiratory chain is essential for electron transfer and the generation of the proton gradient across the inner mitochondrial membrane. Damage to the proteins of the respiratory chain complexes, a common feature in various pathologies, reduces mitochondrial efficiency and cellular energy production [31]. Electron transport chain complex II, or succinate dehydrogenase, is another critical complex that links the tricarboxylic acid cycle to the ETC by transferring electrons from succinate to ubiquinone [46]. Complex II comprises four protein subunits (SDHA, SDHB, SDHC, SDHD) encoded by succinate dehydrogenase (SDH) genes [46]. Its disruption can cause mitochondrial dysfunction, leading to decreased ATP production, increased ROS formation, and ultimately, cardiac damage [47].

Ischemia/reperfusion injury is closely associated with defects in the mitochondrial respiratory chain and other metabolic components, leading to increased ROS production, bioenergetic failure, and cell death. Complex II plays a vital role in coupling mitochondrial respiratory capacity with ETC activity and cell survival during ischemia [46]. Ischemia has been shown to suppress all ETC complexes, with complex I being particularly susceptible. Moreover, complex II disruption significantly impacts cell survival, ROS balance, and cellular bioenergetics—key factors determining tissue viability [48]. In our study, the cytotoxicity inducer H_2_O_2_ decreased the levels of the main subunits of complexes I and II, which would disrupt ETC function and contribute to the observed increase in cytosolic Ca^2+^, ROS production, and loss of mitochondrial potential, culminating in reduced AC16 cardiomyocyte viability. AST prevented the decrease in the subunits of complexes I and II, and under these conditions, cytosolic Ca^2+^, ROS production, and mitochondrial potential remained at control levels. Although DOX also decreased cell viability and ΔΨm while increasing cytosolic Ca^2+^ and ROS, it did not significantly reduce the levels of the examined subunits. This suggests that the mechanisms of action of DOX and H_2_O_2_ are different, and the protective effect of AST against DOX toxicity may involve alternative signaling pathways.

## 5. Conclusions

In this study, we demonstrated that the cytotoxic effects of H_2_O_2_ and DOX in human AC16 cardiomyocytes result in decreased cell viability, increased cytosolic Ca^2+^ levels, elevated ROS production, and loss of mitochondrial membrane potential, collectively indicating severe mitochondrial dysfunction. Pre-incubation with astaxanthin (AST) effectively counteracted these detrimental effects. In the presence of the cytotoxicity inducers, AST reduced cytosolic Ca^2+^ levels and ROS production, while restoring the mitochondrial membrane potential, thereby preventing mitochondrial dysfunction and enhancing cell survival. At the molecular level, H_2_O_2_ treatment increased the levels of caspase-8 and caspase-3, key initiator and effector caspases in apoptosis. AST abolished this increase, even in the presence of H_2_O_2_, underscoring its anti-apoptotic properties. A similar protective effect was observed for the ER stress proteins BIP and CHOP, which were elevated by H_2_O_2_ and normalized by AST pre-treatment. Notably, DOX induced a different protein expression profile compared to H_2_O_2_, particularly by significantly increasing ERO1α levels, which was not observed with H_2_O_2_. This suggests that the two inducers operate through distinct mechanisms. AST was able to mitigate the DOX-induced increase in ERO1α, indicating its ability to modulate this specific pathway. Furthermore, H_2_O_2_, but not DOX, reduced the levels of the main subunits of mitochondrial complexes I (NDUFB8) and II (SDHB). AST pre-treatment completely prevented this H_2_O_2_-induced loss, preserving the integrity of the electron transport chain.

In summary, our findings provide compelling evidence that astaxanthin exerts a potent protective effect against oxidative and chemotherapeutic stress in human cardiomyocytes by preserving mitochondrial function, modulating apoptotic signaling, and alleviating endoplasmic reticulum stress. Based on the results obtained in this and our previous studies, we conclude that AST represents a promising natural compound for the prevention of cardiovascular complications associated with oxidative damage and drug-induced cardiotoxicity. While this study elucidates the multifaceted cardioprotective properties of astaxanthin in stressed cardiomyocytes, these findings constitute a foundational step. Further investigation utilizing more complex physiological models, a deeper mechanistic inquiry, and clinically relevant dosing regimens is essential to fully validate these results and assess their translational potential for therapeutic development.

## Data Availability

The original contributions presented in this study are included in the article. Further inquiries can be directed to the corresponding author.
